# Retraction for Sun et al., “Gut Microbiota Mediates the Therapeutic Effect of Monoclonal Anti-TLR4 Antibody on Acetaminophen-Induced Acute Liver Injury in Mice”

**DOI:** 10.1128/spectrum.03116-22

**Published:** 2022-12-13

**Authors:** Xuewei Sun, Qian Cui, Juan Ni, Xiaoguang Liu, Jin Zhu, Tingting Zhou, HuaYing Huang, Ke OuYang, Yulong Wu, Zhan Yang

## RETRACTION

Volume 10, no. 3, e00647-22, 2022, https://doi.org/10.1128/spectrum.00647-22. We hereby retract this article due to the method for administering the monoclonal anti-TLR4 antibody (ATAB) described in the original paper being inaccurate, as this may influence the readers' understanding of the effect of the ATAB. This inaccuracy was brought to our attention by a Letter to the Editor (https://doi.org/10.1128/spectrum.01863-22). We have responded to the concerns regarding the original paper in our reply to the letter (https://doi.org/10.1128/spectrum.02377-22).

We apologize for any inconvenience caused to the readers.

